# Challenges in the Surgical Treatment of Atrioventricular Septal Defect in Children With and Without Down Syndrome in Romania-A Developing Country

**DOI:** 10.3389/fped.2021.612644

**Published:** 2021-07-07

**Authors:** Ioana-Cristina Olariu, Anca Popoiu, Andrada-Mara Ardelean, Raluca Isac, Ruxandra Maria Steflea, Tudor Olariu, Adela Chirita-Emandi, Ramona Stroescu, Mihai Gafencu, Gabriela Doros

**Affiliations:** ^1^Department of Pediatrics, “Victor Babeş” University of Medicine and Pharmacy, Timisoara, Romania; ^2^Department of Pediatrics, “Louis Turcanu” Emergency Hospital for Children, Timisoara, Romania; ^3^Department of Organic Chemistry, Faculty of Pharmacy, “Victor Babes” University of Medicine and Pharmacy, Timisoara, Romania; ^4^Department of Microscopic Morphology Genetics Discipline, Center of Genomic Medicine Timisoara, “Victor Babeş” University of Medicine and Pharmacy, Timisoara, Romania; ^5^Regional Centre of Medical Genetics Timis, “Louis Turcanu” Emergency Hospital for Children Timisoara, Part of ERN ITHACA, Timisoara, Romania

**Keywords:** atrioventricular septal defect, Down syndrome, congenital heart disease, pulmonary hypertension, risk factors

## Abstract

**Background:** Atrioventricular septal defect (AVSD) is a cardiac malformation that accounts for up to 5% of total congenital heart disease, occurring with high frequency in people with Down Syndrome (DS). We aimed to establish the surgical challenges and outcome of medical care in different types of AVSD in children with DS compared to those without DS (WDS).

**Methods:** The study included 62 children (31 with DS) with AVSD, evaluated over a 5 year period.

**Results:** Complete AVSD was observed in 49 (79%) children (27 with DS). Six children had partial AVSD (all WDS) and seven had intermediate types of AVSD (4 with DS). Eight children had unbalanced complete AVSD (1 DS). Median age at diagnosis and age at surgical intervention in complete AVSD was not significantly different in children with DS compared to those WDS (7.5 months vs. 8.6). Median age at surgical intervention for partial and transitional AVSDs was 10.5 months for DS and 17.8 months in those without DS. A large number of patients were not operated: 13/31 with DS and 8/31 WDS.

**Conclusion:** The complete form of AVSD was more frequent in DS group, having worse prognosis, while unbalanced AVSD was observed predominantly in the group without DS. Children with DS required special attention due to increased risk of pulmonary hypertension. Late diagnosis was an important risk factor for poor prognosis, in the setting of suboptimal access to cardiac surgery for patients in Romania. Although post-surgery mortality was low, infant mortality before surgery remains high. Increased awareness is needed in order to provide early diagnosis of AVSD and enable optimal surgical treatment.

## Introduction

Atrioventricular septal defect (AVSD) or endocardial cushion defect is a cardiac malformation characterized by a variable deficiency of the crux cordis in the developing heart. AVSD accounts for 4–5% of total congenital heart diseases (1). The International Pediatric and Congenital Cardiac Code classified AVSDs into four main groups: complete, partial (or incomplete), intermediate (or transitional), and AVSDs with ventricular imbalance (unbalanced AVSD) (2). Most AVSDs are complete (up to 75%), and account for 3% of all cardiac malformations (2, 3).

Numerous recent studies contributed to a better understanding of genetic causes in congenital heart diseases (4–6). AVSD has been observed in patients with Down syndrome (DS), left or right isomerism, Noonan Syndrome and other (7, 8). Almost half (40–45%) of children with DS have congenital heart diseases. Of these, 35–40% have AVSD (9, 10). The prevalence of complete AVSD is 0.3–0.5% of the populations without DS (WDS). The complete form of AVSD is more frequently associated with additional cardiac defects, left-sided obstructive lesions being more prevalent in children WDS (11, 12). Complete AVSD is observed in almost all patients with right isomerism and in 25% of those with left isomerism (3). The partial forms of AVSD are less frequently associated with chromosomal anomalies (13). A study by Marino Bruno et al. showed that Noonan syndrome is associated with an increased risk for partial AVSD (14). Nonetheless, AVSD can also be non-syndromic (15).

AVSD has major health implications due to the significant interatrial and interventricular systemic-pulmonary shunt leading to increased pressure in the right ventricle with volume overloading, pulmonary hypertension (PAH), heart failure and consequent failure to thrive (16–18). Therefore, precocious diagnosis and surgical treatment is crucial, in order to avoid irreversible complications. Ideally, surgical treatment of complete AVSD should be performed before the age of 6 months (1, 18, 19). In partial and transitional AVSDs surgical treatment may be delayed until the age of 1.6 years or later, if the malformation is well-tolerated (20, 21). Major risk factors for development of PAH in patients with AVSD include presence of DS and age at surgical intervention (3, 17). Because AVSD is common in DS and the onset of PAH in DS is earlier, these patients need special medical management.

We aimed to evaluate the age at diagnosis, age at the time of surgery, and postoperative evolution, in children with AVSD and DS compared to those WDS, in order to understand if specific management is warranted.

## Materials and Methods

The study included 62 children diagnosed with AVSD, admitted in a tertiary clinical care center, the “Louis Turcanu” Emergency Hospital for Children, Timişoara, Romania, between 2014 and 2019. Most children were living in the western Romania, yet some were referred from other regions of the country. We compared children with DS and those WDS, in regards to type of AVSD, age at diagnosis, age at the time of surgery and evolution. AVSDs diagnosed prenatally where termination of pregnancy was chosen were not included in the study.

The echocardiografic examination is the cornerstone for diagnosis and types of AVSDs have been classified according to the International Pediatric and Congenital Cardiac Code as follows: *complete AVSDs* include an ostium primum defect with a non-restrictive inlet ventricular defect and a common atrioventricular valve orifice. *Partial AVSDs* have an isolated ostium primum defect and partitioning of the common atrioventricular valve into two separate atrioventricular orifices (cleft in the mitral valve). *Intermediate or transitional AVSDs* consists of an ostium primum defect in association with a restrictive inlet ventricular defect and two valvular orifices. *Unbalanced AVSD* is defined as malalignment of the atrioventricular valve, being unequal for both ventricles, resulting in one ventricle receiving more blood flow and the other ventricle being underdeveloped (hypoplastic) (2).

The retrospective study included the evaluation of medical records, chest radiography, 12-lead electrocardiogram (ECG), echocardiograms, intraoperative findings, and surgical outcomes of all patients with AVSD. PAH was defined as a pressure gradient estimated by Doppler on tricuspid valve regurgitation over 30 mmHg or a systolic pulmonary artery pressure of more than half systolic systemic pressure. In children presenting severe cardiac failure, blood cardiac biomarkers (troponins and N-terminal pro b-type Natriuretic Peptide NT-pro BNP) were evaluated.

### Statistical Analysis

The cardiac diagnoses were presented in accordance to the classification of the International Pediatric and Congenital Cardiac Code (2). The statistical analysis is descriptive. Data was analyzed using IBM-SPSS version 25 (IBM, Armonk, New York, U.S.A.). Shapiro-Wilk test was used to detect normal distribution. As variables were non-normally distributed, data is presented as median with interquartile range (IQR). Mann-Whitney test was used in continuous variables studied and Chi Square test for categorical variables to evaluate statistical significance of differences between the two groups.

### Ethics

As the study analyzed data that was routinely collected as part of the clinical care of the patient, the study did not require informed consent and was approved as an evaluation of health care delivery. Data were obtained by review of files and electronic medical records. Patient anonymity was preserved throughout.

## Results

Between 2014 and 2019, 62 patients diagnosed with AVSD were evaluated in our tertiary pediatric hospital. Thirty-one children were also diagnosed with DS, while thirty-one were WDS. The male-to-female distribution of AVSD was approximately equal. The male-to female ratio was: in DS 1:1.2 (14:17), in WDS 1.06:1 (16:15). Regarding the types of AVSD, 49 of 62 children had complete AVSD (27 DS, 22 WDS), 6 had partial AVSD (all WDS) and 7 had intermediate AVSD (4 DS, 3 WDS). Among the 49 children with complete AVSD, 8 presented unbalanced ventricles (1 DS, 7 WDS).

Age at diagnosis ranged between 1 day and 15 years (details presented in Table 1). In DS group median age at diagnosis was 7.0 days (IQR, 1 day−31 months) and 10.5 days (IQR, 1 day−7 months) in WDS group, *p* = 0.805 (Mann Whitney). Of all patients, 39 were diagnosed before 6 weeks of age (20 DS, 19 WDS). Eleven patients with AVSD were diagnosed after 1 year of age, 5 with DS (all complete AVSDs), and 6 WDS (2 complete AVSD, 3 partial and 1 transitional AVSD). Only 4 patients were diagnosed with AVSD before birth (1 DS, 3 WDS).

**Table 1:** Type of AVSD and age at diagnosis.

**AVSD type**	**Group DS**		**Group WDS**	
	**No**.	**Age at diagnosis**	**No**.	**Age at diagnosis**
Complete	18 4 5	≤6 weeks 6 weeks−6 months >6 months (range 2.5–14 years)	16 4 2	≤6 weeks 6 weeks−6 months >6 months (range 3–5 years)
Partial	0		1 1 4	≤6 weeks 6 weeks−6 months >6 months (range 3–15 years)
Transitional	2 2	≤ 6 weeks 6 weeks−6 months	2 1	≤6 weeks >6 months (10 years)

*AVSD, Atrioventricular septal defect; DS, Children with Down syndrome; WDS, children without Down syndrome*.

AVSD was associated with other cardiac (presented in Table 2) and extracardiac anomalies (presented in Table 3). In DS group 19 patients (61.3%) were found with other cardiac anomalies, in WDS group 20 (64%). ASD ostium secundum (13 vs. 6), multiple VSD (2 vs. 0), and Tetralogy of Fallot (3 vs. 0) were the most frequent in DS group, while severe cardiac anomalies such as unbalanced ventricles (1 vs. 7), double-outlet right ventricle (0 vs. 7), aortic stenosis (1 vs. 4) were more frequent among patients WDS. None of partial or transitional AVSD had unbalanced ventricles. Left side obstructive lesions were found in two children with partial or transitional AVSD, these being from the group WDS.

**Table 2:** Incidence of other cardiovascular anomalies in 62 patients with atrioventricular septal defect.

	**All Patients**		**Group DS**		**Group WDS**	
**Patients (*n*)**	**No. 62**	**%**	**No. 31**	**%**	**No. 31**	**%**
ASD ostium secundum	19	30.6	13	41.9	6	20.6
PDA	8	12.9	4	12.9	4	13.8
Multiple VSD	2	3.22	2	6.4	0	0
Tetralogy of Fallot	3	4.83	3	9.6	0	0
Double orifice mitral valve	1	1.6	0	0	1	3.44
Aortic stenosis	5	8.06	1	3.22	4	13.8
APVC (total or partial)	2	3.22	0	0	2	6.7
Dextrocardia	2	3.22	0	0	2	6.7
DORV	7	11.3	0	0	7	24.1
TGA	1	1.6	0	0	1	3.44
LPSVC	1	1.6	0	0	1	3.44
Interrupted IVC	2	3.22	0	0	2	6.7

*ASD, Atrial septal defect; PDA, Persistent ductus arteriosus; LPSVC, left persistent superior vena cava; APVC, anomalous pulmonary venous connection; VSD, Ventricular septal defect; DORV, double-outlet right ventricle; TGA, transposition of the great arteries; IVC, inferior vena cava; DS, Children with Down syndrome; WDS, children without Down syndrome*.

**Table 3:** Associated extracardiac anomalies for patients with and without Down syndrome.

**Associated extracardiac anomalies**	**DS**	**WDS**
	**Number of children**	
Digestive system anomaly (5)		
- Duodenal atresia - Annular pancreas - Hirschsprung disease anal atresia	1 1 1 1	0 0 0 1
Urinary anomalies (2) - Hydronephrosis	1	1
Eye anomalies (2) - cataract, ectropion	2	0
Oral clefts (Cleft lip/palate) (1)	0	1
Musculoskeletal system anomalies (4)		
- Dislocation of the hip and patella - Syndactyly - Vertebral abnormalities	2 1 1	0 0 0
Left or right isomerism (8)	0	8
Genital anomalies (1)	0	1
Total	9	12

*DS, Children with Down syndrome; WDS, children without Down syndrome*.

Extracardiac abnormalities were found in 25.8% (8/31) patients from DS group and 32.25% (10/31) patients in those without DS (presented in Table 3). Some of them had multiple malformations. Left or right isomerism was associated with AVSD in 8 patients, all from WDS group. One child diagnosed with AVSD, presented a complex cardiac phenotype (Tetralogy of Fallot) and a rare double aneuploidy (Down and Klinefelter syndrome, 48, XXY, +21 karyotype). He died at 7 months due to pulmonary infection.

At physical examination all children with AVSD presented systolic murmur (grade 2/6 to 6/6). Half of the children with AVSD presented signs of heart failure such as: dyspnea, feeding difficulties, poor growth, tachycardia, tachypnea, subcostal and intercostal retractions, wheezing, hepatic enlargement, poor peripheral blood perfusion, and diaphoresis. Three of them had pulmonary edema: one from DS group and two from the WDS group, all of them were under 6 months of age. Patients with AVSD had feeding problems and presented symptoms in the first few months of life. Failure to thrive was a common finding, 27 patients (43%) had malnutrition (<Percentile 5 on World Health Organization growth references) before surgical correction: 15 from group DS (12 with complete AVSD and 3 with transitional AVSD), 12 WDS (8 with complete AVSD, 2 with partial form and 2 with transitional AVSD).

Electrocardiograms (12-lead) showed sinus rhythm, left-axis-deviation (44/62, 71%), prominent P waves due to atrial enlargement, and signs of possible biventricular hypertrophy. Prolonged PR interval were detected in 6 children at diagnosis, 3 with complete AVSD, 2 partial and 1 transitional AVSD. Chest X-ray showed enlarged cardiac silhouette and increased pulmonary vascular markings in almost all children. The cardiomegaly was mild in partial AVSDs and significant in complete AVSDs.

Echocardiography showed the atrial and ventricular components of the atrioventricular septal defect with medium and large ostium primum defect in 53 children (23 DS, 30 WDS), while 29 patients had large inlet ventricular defect (>6 mm). Of them, 19 were from DS-group (17 with complete AVSD and 2 transitional AVSD) and 10 were from WDS group (8 complete and 2 transitional AVSDs).

PAH before surgery was identified in 34 children with AVSD (56%), 61% from the DS group. Moderate PAH was identified in 31% of children (12 DS, 7 WDS), while 24% (7 DS, 8 WDS) had severe PAH. One patient from DS group that was diagnosed late with AVSD presented Eisenmenger disease. Cardiac catheterization was performed in those with late diagnosis in order to establish the severity of PAH.

Cardiac biomarkers were determined in patients with complex AVSD and heart failure. Half of children with AVSD presented heart failure (16 DS, 18 WDS). Pathological values of NT-pro BNP and troponins were detected in patients with severe cardiac involvement (5 DS, 8 WDS). NT-pro BNP values were elevated, up to 50 times higher than normal, proportional to the degree of heart failure. Values of NT-pro BNP ranged from 207.5 to 17,482 pg/ml with a mean of 4,912 pg/ml.

Of the 62 children with AVSD, 41 had cardiac surgery. Of them, 18 patients were in the DS group and 23 in WDS group (Table 4). Median age at surgical intervention in complete AVSD was 7.5 months in DS group and 8.6 months in WDS (*p* = 0.764). Median age at surgical intervention for partial and transitional AVSDs was 10.5 months (IQR, 5–18 months) for DS and 17.8 months (IQR, 9–19 months) for WDS patients. Ten children (16%) were diagnosed late (6 DS−19.3%, 4 WDS−13.7%). In these children, irreversible PAH was already present. Eleven patients died before surgical intervention, 7 from DS group (22.5%), and 4 from WDS group (13.7%). No deaths were reported in the perioperative period.

**Table 4:** Age at surgery.

**Age at surgery**	**DS (*n* = 18)**	**%**	**WDS (*n* = 23)**	**%**	**Total**
<6 months	10	32.2	8	25.8	18 (29%)
6–12 months	4	12.9	9	31	13 (20.9%)
1–3 years	3	9.6	4	13.7	7 (11.3%)
>3 years	1	3.22	2	6.45	3 (4.8%)
Not operated	13	41.9	8	25.8	21 (33.8%)

*DS, Children with Down syndrome; WDS, children without Down syndrome*.

Postoperative complications following surgical repair of AVSD were: left atrioventricular valve (AV valve) regurgitation, left ventricular outflow tract obstruction, left AV valve stenosis, residual VSD, late-onset complete heart block and pulmonary vascular disease. In the DS group we identified one patient with severe left AV valve insufficiency and DS, who was a candidate for a second surgery (unfortunately pulmonary infection lead to exitus). Another child presented left ventricular outflow tract obstruction. Three patients were left with small residual VSDs with insignificant rest shunt. A child with complete heart block required pacemaker at 12 days after surgery. Severe PAH was identified in 3 children with complete AVSD, one presenting unbalanced ventricles. These patients required endothelin receptor antagonist treatment up to 2 years after surgery.

In the WDS group, 4 children presented severe left AV valve insufficiency (only 1 required corrective surgery after first intervention). Another child had severe left AV valve stenosis that required re-intervention within 1 month after the first intervention. One other child with complete heart block required pacemaker. Only one child had persistent severe PAH after surgery. This child had a complex malformation with unbalanced ventricles, partial anomalous pulmonary venous drainage and heterotaxy syndrome, and he received treatment with phosphodiesterase type 5 inhibitors (sildenafil). Other important postoperative complications in children with complete AVSD were: endocarditis in two children (1 DS, 1 WDS), secondary ischemic stroke after cerebral embolization (a patient with DS, totally recovered) in one patient and protein-losing enteropathy after Fontan intervention (another patient with heterotaxy syndrome, WDS). Partial and transitional AVSDs had good postoperative evolution, without major complications. Postoperative evolution of patients with AVSD was favorable, with the exception of one child with DS, severe PAH and severe left AV valve insufficiency, who died 1 year after surgery (at the age of 2 years).

## Discussion

We aimed to establish if a particular medical management is required in children with DS and AVSD by evaluation of 31 children with DS compared to 31 children without DS, in regards, to type of AVSD, age at diagnosis, age at the time of surgery and postoperative evolution, in a Romanian setting.

The prevalence of complete AVSD was almost 80%, slightly more than in other published reports (2, 3). Complete AVSD was the most common form in both groups. Conversely, partial AVSD was identified only in the group WDS in our cohort, while transitional AVSD were distributed similarly in both groups as showed by other reports (2, 3). Almost 13% of AVSD had unbalanced ventricles, compared to a prevalence of 10% in literature (22, 23). Unbalanced AVSDs are frequently associate with left or right isomerism, required a special approach, and these comorbidities had a major impact on the medical outcome (24–26).

The detection rate of DS in pregnancy may be 90–95%, which leads to an earlier detection of congenital heart diseases, especially of AVSD. In this case, most families choose to termination of pregnancy (9, 27, 28). However, in this cohort, only four children with AVSD were diagnosed during pregnancy and the parents decided to continue the pregnancy. In Romania there are still a large number of pregnant women who do not attend assessment during pregnancy. Nonetheless, our study design did not include an evaluation of cases diagnosed prenatally, where families opted for termination of pregnancy. After birth, the age at diagnosis for AVSD was similar for both groups. Both genders were equally affected.

Age at diagnosis was late for several children in this cohort, despite the recommendations that routine cardiac screening should be performed for all newborn babies with DS. UK and Ireland Down Syndrome Medical Interest Group′s (DSMIG) guidelines recommend that the cardiac status of all children with DS should be established before 6 weeks of age, by clinical, ECG and cardiac ultrasound evaluation (29). Possibly, improved awareness for AVSD complications in primary care is needed, in order to improve referral to tertiary care facilities in Romania.

AVSD has been associated with other heart abnormalities in more than half of children, while extracardiac abnormalities have been found in 25–30% of them, similarly to other reports in literature (27, 30, 31). Digestive system and musculoskeletal anomalies were more frequent in DS group, while isomerism was identified only in the group WDS. Knowledge regarding the existence of extracardiac abnormalities is important as they require a multidisciplinary approach.

The optimal surgical time for complete AVSD is between the 3rd and 6th month of life, before PAH is established (1, 3, 16). Importantly, eleven children in our study died before surgery, more from the DS group, suggesting early diagnosis and follow-up is needed, according to AVSD severity. Most of these cases presented too late at the hospital. There was no difference in surgical technique between AVSD in DS and WDS group (32). Several studies demonstrate that DS patients need reoperation for left AV valve regurgitation later than those WDS (18, 19, 33, 34).

Children with partial and transitional AVSD are largely asymptomatic so referral for surgical repair may be delayed to preschool or older age (20, 21, 35). In a study made by Minich et al. median age at surgery was 1.8 years (21). Patients with partial or transitional AVSDs in our group had no major postoperative complications. Special attention is needed for partial or transitional AVSDs that associates left-side obstructive lesions or unbalanced ventricles, especially those WDS.

Clinical symptoms in AVSD occur in infancy as a result of high pulmonary blood flow associated with PAH (3, 17). Patients with no ventricular component or a small ventricular defect and good AV valve function were asymptomatic in our cohort. Failure to thrive, congestive heart failure and frequent pulmonary infections, were invariably observed children from literature and in this study (3). In selected cases, patients with cardiac failure benefit from determining cardiac biomarkers (36). Pathological values were detected in complex AVSD with severe cardiac involvement in this study.

PAH before surgery was identified in more than 60% from the DS group in this study. After surgery, 3 out of 4 patients that remained with severe PAH had DS, suggesting that special attention is needed in postoperative care. Children with DS and large left-to-right shunt were shown to develop PAH much earlier than children without DS with similar defects (37).

In children with unrepaired complete AVSD, irreversible pulmonary vascular disease becomes increasingly common with age, and affects almost all patients older than 2 years of age (3). A study by Sharma et al. showed that 5 out of 10 children with DS develop PAH and the risk increases to almost 8 out of 10 children with DS and AVSD (37). Early correction of AVSDs can help prevent the development of irreversible pulmonary vascular disease (17). In our study, one patient with Down syndrome presented Eisenmenger syndrome, due to the fact that he was diagnosed late (age 3 years). Early recognition of lesions allows appropriate and timely treatment. Survival following AVSD repair is over 85% at 10 years and over 82% at 20 years after surgery (33, 38). Several studies have showed that the complications risk after AVSD surgical repair in DS are not increased, with the exception of the risk for PAH (16, 18, 39). Life expectancy of patients with DS has increased significantly in developed countries, with a current expected age of 60 years as compared to only 12 years life expectancy in the 1940s (7, 40).

There were several limitations in our study. Our study has a relatively small sample size thus results may not enable accurate identification of significant differences between groups. Therefore, larger studies are needed to further evaluate differences in medical outcome between groups in a similar setting. As several children were diagnosed late in this cohort, the results cannot be generalized for other groups or other countries. Social and educational status of families was not evaluated in this study. Late diagnosis and surgical treatment were caused largely by a particular medical care setting in Romania in former years, which included: low number of pediatric cardiologists and pediatric cardiovascular surgeons, few tertiary pediatric cardiovascular surgery clinics, coupled with low referral rates of newborns to tertiary clinics, as well as family's fear of surgery. Nonetheless, these results are needed to understand morbidity and mortality rates in AVSD, in children with or without DS, in a developing economy. Increased awareness health risks associated with AVSD could bring improved referral rates to tertiary care cardiology clinics.

## Conclusions

The complete form of AVSD was more frequent in DS group, having worse prognosis, while unbalanced AVSD was observed predominantly in the group WDS. Age at diagnosis ranged between 1 day and 15 years. There were no significant differences between the age at diagnosis and age at surgical intervention in children with DS or WDS, however, postoperative care for children with DS required special attention due to risk of PAH. The postoperative results were encouraging; however, a large number of children with AVSD did not receive surgical treatment. Late diagnosis was an important risk factor for poor prognosis, in the setting of suboptimal access to cardiac surgery for patients in Romania. Increased awareness is needed in order to provide early diagnosis of AVSD and enable optimal surgical treatment, before occurrence of irreversible PAH. This way, both increased life expectancy and increased quality of life is expected for these people.

## Data Availability Statement

The raw data supporting the conclusions of this article will be made available by the authors, without undue reservation.

## Ethics Statement

Ethical review and approval was not required for the study on human participants in accordance with the local legislation and institutional requirements. Written informed consent to participate in this study was provided by the participants' legal guardian/next of kin.

## Author Contributions

I-CO, GD, and AP: conceptualization. AP and RS: methodology. AC-E and TO: software. GD, AC-E, and AP: validation. GD and I-CO: formal analysis. I-CO, A-MA, RI, and RS: investigation. AP and GD: resources. A-MA, RI, RMS, and TO: data curation. I-CO: writing. GD, AP, and MG: supervision. All authors have read and agreed to the published version of the manuscript.

## Conflict of Interest

The authors declare that the research was conducted in the absence of any commercial or financial relationships that could be construed as a potential conflict of interest.
